# The network approach to psychopathology: investigating inter-individual variability and the association with clinical relapse in psychosis

**DOI:** 10.1038/s41537-025-00636-8

**Published:** 2025-07-03

**Authors:** George Gillett, Dan W. Joyce, Cedric E. Ginestet, James H. MacCabe, Nicholas Meyer

**Affiliations:** 1 Centre for Family Research, Department of Psychology, University of Cambridge, Free School Lane, Cambridge, UK; 2 Department of Primary Care and Mental Health, University of Liverpool, Liverpool, UK; 3 Department of Biostatistics and Health Informatics, King’s College London, London, UK; 4 Department of Psychosis Studies, Institute of Psychology, Psychiatry and Neuroscience, King’s College London, London, UK; 5 Insomnia and Behavioural Sleep Medicine Clinic, University College London Hospitals NHS Foundation Trust, London, UK

**Keywords:** Psychosis, Schizophrenia, Human behaviour

## Abstract

Recent years have seen a proliferation of interest in psychological networks, which conceptualise psychopathology as networks of inter-connected, mutually reinforcing symptoms. It has been hypothesised that the topological structure of such networks is associated with clinical presentation. Analysing data from a longitudinal study of participants diagnosed with psychosis, we identify substantial inter-individual variability in network structure, problematising causal inference from cross-sectional networks. Additionally, we do not find strong evidence for an association between network structure and clinical relapse.

## Introduction

In contrast to traditional models of psychopathology which conceptualise symptoms arising from a common latent cause, the network approach to psychopathology suggests that mental disorders arise from interactions between mutually reinforcing symptoms^1^. Network theorists therefore emphasise the importance of the relationships between symptoms in the emergence and persistence of psychiatric distress (such as worry, rumination and anhedonia in depression). The field therefore offers a promising conceptual and methodological framework to study psychiatric disorders, without relying on assumptions about hidden, latent entities.

Using principles derived from graph theory, network approaches typically derive centrality measures from a partial correlation network to assess a symptom’s apparent role – and relative importance - within the psychological network. Centrality measures include strength, closeness and betweenness (Supplementary Item A)^2^. It is theorised that the overall network structure may also predispose towards mental ill-health through the concept of hysteresis - the proposal that densely connected symptom networks more readily precipitate and perpetuate psychopathology compared to sparsely connected networks^3^. Psychosis may be a useful context in which to study the concept of hysteresis, given that relapse comprises many different cognitive and affective symptoms, while relapse status can often be reliably ascertained by clinical assessment.

Recent years have seen a proliferation of interest in network psychopathology^4,5^. Although methods vary, network analyses most commonly comprise large cross-sectional datasets of psychometric data. Centrality measures derived from such networks are often used to infer causal associations between symptoms^4,5^. For instance, cross-sectional network analysis of psychotic symptoms have been used to draw conclusions about psychological mechanisms and putative treatment targets^6,7^. However, the field has received criticism for failing to confirm necessary assumptions prior to embarking upon causal inference^5^. One such assumption is that network structure should be stable across individuals if within-individual interpretations are to be inferred from cross-sectional network data. This assumption has not been robustly tested, bringing into question whether such networks represent mechanistic associations between symptoms, or simply represent the clustering of symptoms within a population.

Using data from a rich longitudinal dataset, we test this assumption by assessing the extent to which network structure is consistent between individuals diagnosed with a psychotic disorder. We then assess whether the networks of relapsing individuals differ to those of non-relapsing individuals, to examine the theory of hysteresis.

## Methods

### Participants

Data were taken from the Sleepsight project, a one-year longitudinal study of individuals with schizophrenia-spectrum disorders in which participants self-reported symptom severity^8^. Six participants whose data exhibited minimal variation or stereotyped responses, or who contributed less than 100 days of data, were manually excluded from this analysis, to achieve network stability. We present data from 30 participants (11 females; mean age: 41.9 [SD: 8.4] years) diagnosed with either schizophrenia (20), schizoaffective disorder (7) or first-episode psychosis (3). Further clinical and demographic details are given in Supplementary Item B. Participants contributed a mean of 265.3[114.3] days of data across the study.

### Measures

Participants completed a once-daily symptom diary using a smartphone application, consisting of 14 items rated on a seven-point Likert scale, based on previous literature (Supplementary Item C)^9^. The diary asked to what extent participants had been feeling or experiencing the following symptoms: ‘anxious’, ‘irritable’, ‘sad’, ‘stressed’, ‘cheerful’, ‘relaxed’, ‘in control’, ‘suspicious’, ‘trouble concentrating’, ‘preoccupied by thoughts’, ‘others dislike me’, ‘confused’, ‘others influence my thoughts’ and ‘unusual sights and sounds’. A fixed sampling schedule prompted users to complete the symptom diary between 11:00 to 14:00. Detailed study design has been reported previously^8^.

Relapse status was ascertained by weekly review of participants’ electronic health record. Any deterioration resulting in a change in management (including change in medication, escalation of community input or hospital admission) was classified as relapse. Twelve participants experienced at least one relapse during the period (three participants experienced more than one relapse event).

### Data analysis

Positive self-report items were reverse-coded, such that higher scores corresponded to symptom severity. The *goldbricker* function was used to identify redundant items, consistent with other network studies^10^; the ‘relaxed’ item was removed due to collinearity with the ‘stressed’ item. The coding language R was used for all data analysis^11^.

**Fig. 1:**
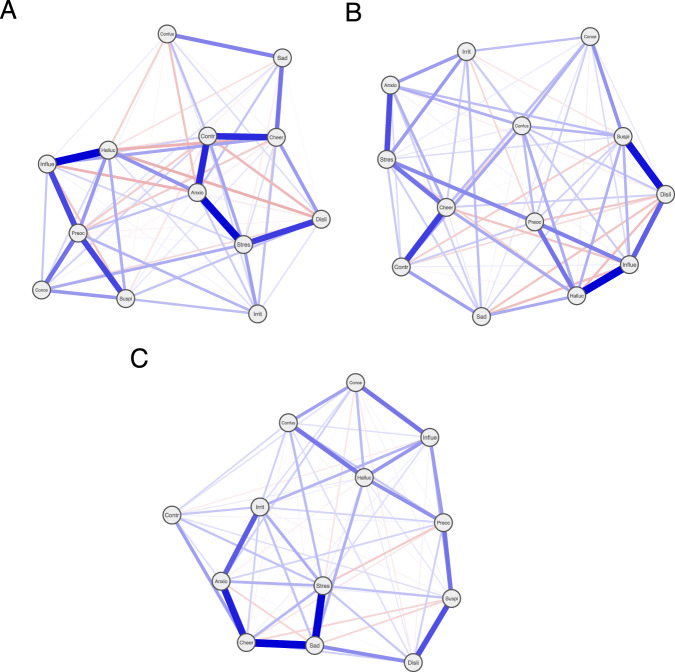
Individual participant networks. Three participants’ networks (**A**–**C**). Each node represents a symptom; each edge represents the partial correlation coefficient between symptoms. Node position is determined by the Fruchterman-Reingold Algorithm. ‘Anxio’ - anxious, ‘Cheer’ - cheerful, ‘Conce’ - trouble concentrating, ‘Confus’ - confused, ‘Contr’ - in control, ‘Disli’ - others dislike me, ‘Halluc’ - unusual sights and sounds, ‘Influe’ - others influence my thoughts, ‘Irrit’ - irritable, ‘Preoc’ - preoccupied by thoughts, ‘Stres’ - stressed, ‘Suspi’ - suspicious.

A non-regularized, weighted, partial correlation network was generated for each participant’s data, consistent with previous literature (Fig. 1)^12^. In such networks, each node represents a symptom and each edge weight represents the partial correlation between two symptoms; higher edge weights confer lower ‘shortest paths’ between nodes. We derived the following centrality indices per symptom for each participant: *strength* (sum of edge weights a node is connected to), *closeness* (reciprocal of the sum of shortest paths between a node and all the other nodes in the network) and *betweenness* (number of shortest paths between symptoms that a given node lies on). We also derived the *average shortest path length* and *global efficiency* for each network, measures of network density.

To assess the extent to which network structure is consistent between individuals diagnosed with a psychotic disorder, we plot and report the standard deviation of each centrality index for each symptom across the entire study sample. To assess whether relapse is associated with network structure, we compare network metrics between the relapse and non-relapse group, using two-sample t-tests and Wilcoxon rank-sum tests. Raw and rank indices were tested to assess whether either the absolute or relative centrality index was associated with clinical relapse. P-values below 0.05 were considered significant. We applied the Benjamini–Hochberg correction for multiple comparisons to control the false discovery rate (FDR) at ≤ 0.10.

## Results

### Inter-individual variability in network structure

**Fig. 2:**
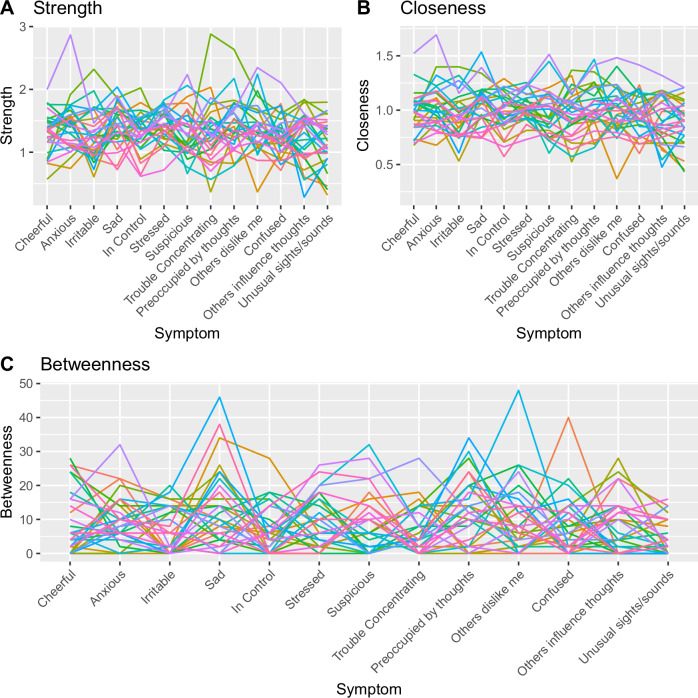
Centrality indices per symptom. Each coloured line represents a different participant’s data. Each plot presents a different centrality index: (**A**): Strength, (**B**): Closeness, (**C**): Betweenness. Closeness is scaled by 10^2^ for ease of interpretation.

Figure 2 shows high inter-individual variability in centrality indices across all symptoms, with no discernible pattern replicated between participants. The standard deviation also indicated high inter-individual variability in centrality indices across symptoms (Supplementary Items D & E).

### Association with clinical presentation

Twelve participants experienced a combined total of 15 relapse events. Relapse was associated with more central cheerfulness (strength; *p* = 0.024, betweenness; *p* = 0.002), and less central ‘in control’ (strength; *p* = 0.019) and ‘trouble concentrating’ (betweenness; *p* = 0.009) symptoms (Supplementary Item E). When ordinal rank indices were compared, relapse was associated with more central cheerfulness (strength; *p* = 0.003, closeness; *p* = 0.008, betweenness; *p* = 0.028) and anxiety (strength; *p* = 0.03, closeness; *p* = 0.016), and less central feelings of ‘others dislike me’ (betweenness; *p* = 0.007; Supplementary Item F). However, none of these results were significant after correction for multiple comparisons at FDR ≤ 0.10. The more central symptoms (cheerfulness and anxiety) also exhibited greater overall (SD), but not day-to-day (Root Mean Square of Successive Differences; RMSSD) symptom variability in the relapse group (Supplementary Item G). However, these differences were not significant after correction for multiple comparisons at FDR ≤ 0.10.

There was no difference in average shortest path length (9.15 vs 8.75; *p* = 0.45) or global efficiency (0.66 vs 0.70; *p* = 0.35) between relapsers and non-relapsers (Supplementary Item E).

## Discussion

Applying conventional network approaches to longitudinal symptom data from 30 participants diagnosed with psychosis, we identify substantial inter-individual variability in network structure. This questions the frequent practice of inferring intra-individual psychological mechanisms from inter-individual cross-sectional data^4,5^. Our empirical findings agree with the caution expressed by other authors, who suggest that patterns amongst symptoms emergent at the level of the group may not hold at the level of the individual^13^. We provide empirical evidence to substantiate this theoretical concern, given it remains a matter of active debate, with some researchers disputing its significance^14^.

We found no strong evidence in support of hysteresis; neither average shortest path length nor global efficiency was associated with relapse. Regarding other group differences, the increased centrality of positive affect and anxiety in the relapse group is interesting, and may warrant further investigation if replicated in other studies. However, the significance of these findings did not survive correction for multiple comparisons. It is also possible that these findings represent artefacts arising from increased variability of these symptoms during psychiatric distress, given that both symptoms also exhibited increased variability in the relapse group^15^. Future studies should present measures of symptom variability alongside centrality metrics.

There are many plausible explanations why network structure may not be stable across participants. True variability may exist in psychological mechanisms between individuals diagnosed with the same condition (in which case network structure is effectively decoupled from clinically meaningful presentation), or network findings may represent artefacts arising from misguided assumptions about how psychological symptoms relate to one another^2^. Our findings are in keeping with a growing literature suggestive of inconsistent findings between network psychopathology studies^5,16^. Novel methodological advances, including the use of time-series networks, may offer promising, more appropriate avenues to assess questions related to psychopathology at the level of an individual^17–19^.

### Limitations

Our sample may have been under-powered to detect an association with relapse, although there is little precedent for determining power in network studies. This study’s relatively small sample size is typical of remote monitoring projects in this patient population, especially for studies which aim to recruit participants for periods long enough to observe clinically meaningful relapse. Furthermore, the clinical utility of any undetected association may be questionable, given our dataset featured high frequency symptom data of one year’s duration. Nonetheless, these findings should be considered exploratory until replicated in an independent dataset. It is possible that our approach of combining longitudinal data into a single network diluted any effect associated with acute clinical relapse; future studies may derive networks for shorter time periods, which would require higher frequency data to achieve network stability. We did not include data on context (such as location or social interactions), which may offer useful insights into how symptoms interact with environmental factors^20^.

## Conclusion

In the first study to empirically assess the network theory of hysteresis using real-world clinical data to our knowledge, we find no strong evidence in support of an association between clinical relapse and global network structure. Furthermore, we identify substantial inter-individual variability in network metrics among participants diagnosed with psychosis. This suggests that network methodology reliant upon partial correlation coefficients obtained from cross-sectional data may be better suited to population-level nosological analysis of phenotype clusters rather than inference about individual psychopathology.

## Supplementary information

Supplementary Material

## Data Availability

The data that support the findings of this study are available on request from the senior author [N.M.]. The data are not publicly available due to them containing information that could compromise research participant privacy/consent.

## Code availability

The code used to generate the findings of this study are available on request from the corresponding author [G.G.].
