# Supplementary material for: The network approach to psychopathology: investigating inter-individual variability and the association with clinical relapse in psychosis
Source: Schizophrenia (Heidelb). 2025 Jul 3;11(1):94. doi: 10.1038/s41537-025-00636-8 (PMC12229628; doi:10.1038/s41537-025-00636-8)
Supplement: Supplementary file 1 — Supplementary Material [file 41537_2025_636_MOESM1_ESM.docx]

**Supplementary Material**

The network approach to psychopathology: investigating inter-individual variability and the association with clinical relapse in psychosis

George Gillett^1*^, Dan W. Joyce^2^, Cedric E. Ginestet^3^, James H. MacCabe^4^, Nicholas Meyer^4,5^

^1^ Centre for Family Research, Department of Psychology, University of Cambridge, Free School Lane, Cambridge, CB2 3RQ

^2^ Department of Primary Care and Mental Health, University of Liverpool, Liverpool, UK

^3^ Department of Biostatistics and Health Informatics, King’s College London, London SE5 8AF, United Kingdom

^4^ Department of Psychosis Studies, Institute of Psychology, Psychiatry and Neuroscience, King’s College London, London

^5^ Insomnia and Behavioural Sleep Medicine Clinic, University College London Hospitals NHS Foundation Trust, London, UK.

*** Correspondence:**Dr George Gillett
gg555@cam.ac.uk

**Supplementary Item A: Summary of network centrality indices**

| Measure | Description |
| --- | --- |
| Strength | Defined as the sum of the absolute edge weights directly connected to a node. This represents a measure of a symptom’s correlation to all the symptoms it is directly associated with. |
| Closeness | Defined as the average shortest path (including indirect paths via other nodes) from a given node to all other nodes in the network. Unlike strength, closeness represents both direct and indirect proximity to all the other nodes in the network. |
| Betweenness | Provides a measure of how often a given node lies on the shortest path between two other nodes, when the routes between all pairs of nodes in the network are considered. Nodes with high betweenness are often assumed to ‘gate-keep’ the relationship between other nodes, whose shortest path they lie on. |

**Supplementary Item B: Clinical and demographic information**

The following antipsychotics were prescribed: clozapine (8), olanzapine (8), aripiprazole (8), risperidone (2), paliperidone (1), quetiapine (1), penfluridol (1). One participant was not prescribed a regular antipsychotic. The following mood stabilisers were prescribed: lamotrigine (3), valproate, lithium. The following antidepressants were prescribed: sertraline (8), fluoxetine (3), venlafaxine (2), citalopram (1), escitalopram (1), paroxetine (1), mirtazapine (1). SCZ: schizophrenia, SAD: schizoaffective disorder, FEP: first-episode psychosis. The Positive and Negative Syndrome Scale (PANSS) and Beck Depression Inventory (BDI) were assessed at baseline.

|  | **Relapse group (n=12)** | **Non-relapse group (n=18)** | **p-value** |
| --- | --- | --- | --- |
| Gender (% female) | 3 (25%) | 8 (44.4%) | - |
| Mean age (SD) | 40.9 (8.1) | 42.6 (8.8) | 0.605 |
| Diagnosis | 5 SCZ; 5 SAD; 2 FEP | 15 SCZ; 2 SAD; 1 FEP | - |
| Mean PANSS (SD) | 71.3 (18.1) | 73.5 (17.2) | 0.746 |
| Mean BDI (SD) | 17.9 (8.2) | 22.1 (13.0) | 0.288 |
| Prescribed mood stabiliser (%) | 2 (16.7%) | 3 (16.7%) | - |
| Prescribed antidepressant (%) | 8 (66.7%) | 9 (50%) | - |

**Supplementary Item C: Self-report EMA items in the Sleepsight study**

Each item was rated on a 7-point Likert scale from ‘Not at all’ to ‘Very Much’

Over the past 24 hours, I have been feeling:

Cheerful

Anxious

Relaxed

Irritable

Sad

In control

Stressed

Over the past 24 hours, I have been experiencing/feeling:

Suspicious

Trouble Concentrating

Preoccupied by Thoughts

Others Dislike Me

Confused

Others Influence My Thoughts

Unusual Sights and Sounds

**Supplementary Item D: Centrality rank indices**

Each coloured line represents a different participant’s data. Each plot presents a different centrality index by ordinal rank: A: Strength, B: Closeness, C: Betweenness.

**Supplementary Item E: Network metrics including centrality indices and average shortest path length**

P-value derived from two-sample t-testing between the relapse and non-relapse groups. Closeness is scaled by 10^2^ for ease of interpretation. * denotes p<0.05. No results were significant after Benjamini–Hochberg correction with FDR at ≤ 0.10 (applied to the family of analyses presented in Supplementary Items E & F).

|  |  | **Mean (SD)** | | | **p-value** |
| --- | --- | --- | --- | --- | --- |
|  |  | **Total cohort (n=30)** | **Relapse group (n=12)** | **Non-relapse group (n=18)** |  |
| **Strength** | **Anxious** | 1.426 (0.395) | 1.549 (0.476) | 1.344 (0.318) | 0.206 |
|  | **Irritable** | 1.280 (0.386) | 1.239 (0.377) | 1.307 (0.400) | 0.642 |
|  | **Sad** | 1.451 (0.324) | 1.370 (0.373) | 1.505 (0.286) | 0.302 |
|  | **Stressed** | 1.427 (0.262) | 1.423 (0.320) | 1.429 (0.226) | 0.955 |
|  | **Cheerful** | 1.316 (0.313) | 1.477 (0.306) | 1.210 (0.276) | 0.024 * |
|  | **In control** | 1.318 (0.312) | 1.147 (0.328) | 1.433 (0.249) | 0.019 * |
|  | **Suspicious** | 1.307 (0.374) | 1.303 (0.476) | 1.309 (0.303) | 0.968 |
|  | **Trouble concentrating** | 1.304 (0.485) | 1.157 (0.328) | 1.402 (0.554) | 0.141 |
|  | **Preoccupied by thoughts** | 1.426 (0.407) | 1.403 (0.366) | 1.441 (0.442) | 0.803 |
|  | **Others dislike me** | 1.390 (0.406) | 1.273 (0.386) | 1.469 (0.410) | 0.198 |
|  | **Confused** | 1.258 (0.316) | 1.233 (0.381) | 1.274 (0.275) | 0.756 |
|  | **Others influence my thoughts** | 1.247 (0.399) | 1.146 (0.357) | 1.315 (0.421) | 0.247 |
|  | **Unusual sights and sounds** | 1.150 (0.373) | 1.090 (0.296) | 1.190 (0.420) | 0.453 |
| **Closeness** | **Anxious** | 1.037 (0.206) | 1.071 (0.247) | 1.015 (0.178) | 0.503 |
|  | **Irritable** | 0.943 (0.203) | 0.912 (0.212) | 0.963 (0.200) | 0.520 |
|  | **Sad** | 1.049 (0.186) | 0.983 (0.190) | 1.093 (0.176) | 0.123 |
|  | **Stressed** | 1.021 (0.124) | 1.023 (0.162) | 1.020 (0.096) | 0.964 |
|  | **Cheerful** | 0.986 (0.180) | 1.053 (0.215) | 0.941 (0.142) | 0.130 |
|  | **In control** | 0.980 (0.175) | 0.918 (0.210) | 1.021 (0.139) | 0.150 |
|  | **Suspicious** | 0.976 (0.213) | 0.961 (0.286) | 0.986 (0.157) | 0.786 |
|  | **Trouble concentrating** | 0.959 (0.210) | 0.891 (0.170) | 1.004 (0.226) | 0.131 |
|  | **Preoccupied by thoughts** | 1.019 (0.208) | 1.009 (0.239) | 1.025 (0.192) | 0.847 |
|  | **Others dislike me** | 0.995 (0.218) | 0.972 (0.210) | 1.010 (0.228) | 0.643 |
|  | **Confused** | 0.962 (0.181) | 0.920 (0.226) | 0.989 (0.145) | 0.360 |
|  | **Others influence my thoughts** | 0.923 (0.219) | 0.895 (0.216) | 0.941 (0.226) | 0.579 |
|  | **Unusual sights and sounds** | 0.885 (0.200) | 0.856 (0.184) | 0.904 (0.213) | 0.518 |
| **Betweenness** | **Anxious** | 9.867 (7.555) | 11.667 (8.773) | 8.667 (6.615) | 0.326 |
|  | **Irritable** | 7.000 (6.762) | 5.333 (6.286) | 8.111 (7.012) | 0.268 |
|  | **Sad** | 13.467 (11.626) | 12.167 (11.424) | 14.333 (12.005) | 0.622 |
|  | **Stressed** | 9.600 (7.673) | 9.833 (8.111) | 9.44 (7.602) | 0.896 |
|  | **Cheerful** | 9.533 (9.580) | 16.333 (9.604) | 5.0 (6.517) | 0.002 * |
|  | **In control** | 6.667 (7.150) | 5.167 (7.004) | 7.667 (7.268) | 0.355 |
|  | **Suspicious** | 8.733 (8.796) | 10.167 (9.590) | 7.778 (8.371) | 0.490 |
|  | **Trouble concentrating** | 6.800 (6.759) | 3.333 (3.651) | 9.111 (7.427) | 0.009 * |
|  | **Preoccupied by thoughts** | 11.933 (9.889) | 14 (9.303) | 10.556 (10.285) | 0.350 |
|  | **Others dislike me** | 10.400 (10.614) | 9.333 (0.432) | 11.111 (11.545) | 0.648 |
|  | **Confused** | 7.933 (8.749) | 5.5 (5.792) | 9.556 (10.095) | 0.174 |
|  | **Others influence my thoughts** | 8.200 (8.277) | 6.333 (7.667) | 9.444 (8.645) | 0.311 |
|  | **Unusual sights and sounds** | 4.400 (4.994) | 4.667 (5.614) | 4.222 (4.697) | 0.823 |
| **Average shortest path** | | 8.906 (1.308) | 9.146 (1.538) | 8.745 (1.149) | 0.450 |
| **Global Efficiency** | | 0.684 (0.133) | 0.656 (0.133) | 0.703 (0.133) | 0.353 |

**Supplementary Item F: Centrality network indices; ordinal rank analyses**

P-value derived from Wilcoxon rank-sum tests between the relapse and non-relapse groups. * denotes p<0.05. No results were significant after Benjamini–Hochberg correction with FDR at ≤ 0.10 (applied to the family of analyses presented in Supplementary Items E & F).

|  |  | **Mean rank (IQR)** | |  |
| --- | --- | --- | --- | --- |
|  |  | **Relapse group (n=12)** | **Non-relapse group (n=18)** | **p-value** |
| **Strength** | **Anxious** | 9.5 (4.5) | 6.722 (5) | 0.030 * |
|  | **Irritable** | 6.333 (6.5) | 6.111 (7.75) | 0.848 |
|  | **Sad** | 8.333 (7.5) | 8.778 (5.75) | 0.949 |
|  | **Stressed** | 8.917 (4) | 8.5 (4.75) | 0.639 |
|  | **Cheerful** | 9.333 (4.25) | 5.5 (3) | 0.003 * |
|  | **In control** | 5.333 (6.5) | 7.667 (3.5) | 0.101 |
|  | **Suspicious** | 7.25 (7.5) | 6.444 (7.5) | 0.595 |
|  | **Trouble concentrating** | 4.667 (2.5) | 7.222 (5.75) | 0.093 |
|  | **Preoccupied by thoughts** | 8.417 (4) | 7.556 (8.75) | 0.831 |
|  | **Others dislike me** | 7.083 (3.5) | 8.167 (6.5) | 0.268 |
|  | **Confused** | 5.25 (4.5) | 5.722 (5.25) | 0.832 |
|  | **Others influence my thoughts** | 5.417 (5.5) | 7.167 (5) | 0.241 |
|  | **Unusual sights and sounds** | 5.167 (3) | 5.444 (4.75) | 0.831 |
| **Closeness** | **Anxious** | 10 (2.75) | 6.889 (4.5) | 0.016 * |
|  | **Irritable** | 5.25 (4.5) | 6.222 (7.25) | 0.551 |
|  | **Sad** | 7.75 (6.25) | 8.556 (5.75) | 0.718 |
|  | **Stressed** | 9 (3.25) | 7.833 (3) | 0.248 |
|  | **Cheerful** | 9.75 (3.75) | 5.722 (6.25) | 0.008 * |
|  | **In control** | 5.75 (7.25) | 7.389 (4.75) | 0.297 |
|  | **Suspicious** | 7.25 (7.25) | 6.722 (7.5) | 0.798 |
|  | **Trouble concentrating** | 4.167 (2) | 7.444 (7.75) | 0.058 |
|  | **Preoccupied by thoughts** | 7.667 (4.75) | 7.889 (4) | 0.815 |
|  | **Others dislike me** | 8 (3.25) | 7.722 (7.5) | 0.915 |
|  | **Confused** | 5.5 (5.25) | 7.111 (4.75) | 0.337 |
|  | **Others influence my thoughts** | 5.833 (4) | 6.222 (7.75) | 0.831 |
|  | **Unusual sights and sounds** | 5.083 (5) | 5.278 (6) | 0.949 |
| **Betweenness** | **Anxious** | 3.667 (2.75) | 4.833 (5) | 0.413 |
|  | **Irritable** | 4.25 (6.25) | 2.667 (2.75) | 0.261 |
|  | **Sad** | 5.667 (3) | 4.167 (4) | 0.069 |
|  | **Stressed** | 5.083 (4.5) | 4.111 (2.75) | 0.316 |
|  | **Cheerful** | 5.833 (2) | 3.611 (5) | 0.028 * |
|  | **In control** | 2.917 (2.25) | 4.833 (4) | 0.055 |
|  | **Suspicious** | 2.5 (1.25) | 4.5 (5.25) | 0.062 |
|  | **Trouble concentrating** | 4.083 (6) | 4.944 (3.75) | 0.428 |
|  | **Preoccupied by thoughts** | 4.75 (2.5) | 3.222 (4) | 0.082 |
|  | **Others dislike me** | 3.25 (3.25) | 5.944 (3.75) | 0.007 * |
|  | **Confused** | 3.5 (3.25) | 4.5 (3.75) | 0.315 |
|  | **Others influence my thoughts** | 2.333 (2.25) | 3.666 (3) | 0.076 |
|  | **Unusual sights and sounds** | 3.5 (4.5) | 3.167 (5) | 0.693 |

**Supplementary Item G: Symptom mean and variability, by relapse group**

P-value derived from two-sample t-testing between the relapse and non-relapse groups. No results were significant after Benjamini–Hochberg correction with FDR at ≤ 0.10 (applied to the family of analyses presented in Supplementary Item G).

|  | **Mean** | | |
| --- | --- | --- | --- |
|  | **Relapse group (n=12)** | **Non-relapse group (n=18)** | **p-value** |
| **Anxious** | 3.852 | 3.270 | 0.234 |
| **Irritable** | 2.775 | 2.894 | 0.813 |
| **Sad** | 3.014 | 3.213 | 0.705 |
| **Stressed** | 3.938 | 3.467 | 0.352 |
| **Cheerful** | 3.075 | 3.167 | 0.836 |
| **In control** | 2.965 | 2.317 | 0.176 |
| **Suspicious** | 3.604 | 3.404 | 0.733 |
| **Trouble concentrating** | 3.717 | 3.279 | 0.440 |
| **Preoccupied by thoughts** | 4.098 | 3.622 | 0.400 |
| **Others dislike me** | 3.376 | 3.497 | 0.845 |
| **Confused** | 2.616 | 2.989 | 0.460 |
| **Others influence my thoughts** | 2.744 | 2.956 | 0.738 |
| **Unusual sights and sounds** | 2.751 | 2.485 | 0.696 |
|  | **Standard Deviation** | | |
| **Anxious** | 1.184 | 0.910 | 0.041 * |
| **Irritable** | 1.069 | 0.973 | 0.461 |
| **Sad** | 1.118 | 0.989 | 0.351 |
| **Stressed** | 1.233 | 1.025 | 0.156 |
| **Cheerful** | 1.094 | 0.827 | 0.028 * |
| **In control** | 0.927 | 0.982 | 0.730 |
| **Suspicious** | 0.876 | 0.983 | 0.617 |
| **Trouble concentrating** | 0.984 | 0.963 | 0.863 |
| **Preoccupied by thoughts** | 1.030 | 0.961 | 0.629 |
| **Others dislike me** | 1.073 | 1.045 | 0.902 |
| **Confused** | 0.929 | 0.882 | 0.722 |
| **Others influence my thoughts** | 0.702 | 0.784 | 0.658 |
| **Unusual sights and sounds** | 0.766 | 0.699 | 0.739 |
|  | **Root mean square of successive differences** | | |
| **Anxious** | 1.060 | 1.049 | 0.950 |
| **Irritable** | 1.068 | 1.131 | 0.672 |
| **Sad** | 1.062 | 1.075 | 0.928 |
| **Stressed** | 1.213 | 1.154 | 0.762 |
| **Cheerful** | 0.966 | 0.974 | 0.949 |
| **In control** | 0.988 | 1.034 | 0.776 |
| **Suspicious** | 0.835 | 1.003 | 0.409 |
| **Trouble concentrating** | 0.973 | 1.059 | 0.557 |
| **Preoccupied by thoughts** | 0.951 | 0.983 | 0.796 |
| **Others dislike me** | 0.851 | 0.922 | 0.706 |
| **Confused** | 0.862 | 0.976 | 0.394 |
| **Others influence my thoughts** | 0.641 | 0.837 | 0.210 |
| **Unusual sights and sounds** | 0.702 | 0.679 | 0.891 |
